# Cytologic-Radiologic Correlation Using Transthoracic CT-Guided FNA for Lung and Mediastinal Masses: Our Experience

**DOI:** 10.1155/2014/343461

**Published:** 2014-11-25

**Authors:** Sanjay Piplani, Rahul Mannan, Monika Lalit, Mridu Manjari, Tejinder S. Bhasin, Jasmin Bawa

**Affiliations:** ^1^Department of Pathology, Sri Guru Ram Das Institute of Medical Sciences & Research, 24 Lane No. 5, Gopal Nagar, Majitha Road, Amritsar, Punjab 143001, India; ^2^Department of Anatomy, Sri Guru Ram Das Institute of Medical Sciences & Research, Amritsar, Punjab, India

## Abstract

*Background and Objectives.* Thoracic lesions account for various benign and malignant conditions. Of these lung carcinoma (mainly primary) is the most common carcinoma in the world. The present study was undertaken to know the pathological spectrum of thoracic lesions and to correlate cytoradiological findings.* Materials and Methods.* The present study was conducted in a tertiary care center of North India on 74 patients over an 18-month period. CT guided transthoracic FNAC (TTFNA) was carried out, and aspirates were drawn, examined, and compared with radiological diagnoses.* Results. *The diagnostic accuracy for FNA in the present study was calculated to be 95.94% (using cytology as the gold standard). The predominant lesion was malignancy (85.1%), followed by suspicions of malignancy and inflammatory pathology (5.40% each). By cytology, the most common malignant lesion was adenocarcinoma (48%) followed by squamous cell carcinoma (40%), small cell carcinoma (8%), and undifferentiated carcinoma (4%). Cytoradiological correlation was found to be 89.2% in the present study.* Conclusion.* Present study thus concludes that TT FNA of thoracic lesions is a simple, safe, economically prudent technique associated with low morbidity and leading to quick and early diagnosis.

## 1. Introduction

Thoracic lesions include a large variety of benign and malignant conditions of lung, pleura, mediastinum, and vertebrae. Primary lung carcinoma is the most common carcinoma in the world today, comprising 12.6% of all the cancers and 17.8% of all the cancer deaths [1]. Lung is also a well-known site for metastatic tumors. In addition, the mediastinum can be involved with a variety of benign lesions as well as by primary and metastatic malignant tumor, many of which present as mediastinal masses [2, 3]. Although clinical data, location, and radiological findings can narrow down the diagnostic possibilities, cytological diagnosis is warranted before initiating the specific treatment for malignant diseases [4].

Percutaneous CT-guided transthoracic fine needle aspiration cytology (TTFNA) is a well-established diagnostic method used in cytological evaluation of thoracic mass lesions. Currently TTFNA for lesions of the lungs and mediastinum is a widely practiced method, where the facilities of standard imaging techniques and cytopathology are available. This procedure provides a safe, rapid, and accurate diagnosis in patients having thoracic mass lesions [3–5]. In patients with lung cancer which is inoperable owing to local factors or the patient's general condition, FNAC confirms the diagnosis and reveals the tumor type [6].

The purpose of the present study was to know the pathological spectrum of thoracic lesions and to correlate the radiological findings with cytological findings obtained from CT-guided FNAC.

## 2. Material and Methods

The present study was conducted on 74 patients who had thoracic mass lesions that were suspected to be neoplastic in most of the cases by chest radiographs and CT scan in the department of pathology, SGRDIMSR, Amritsar, from June 2010 to December 2011.

The procedure was carried out as an outpatient procedure in all the selected patients after explaining the risks and benefits. Informed consent was also obtained from every case. Detailed personal (especially tobacco habit) and occupational history was also taken.

Proper aseptic care was taken by cleaning the skin surface with povidone iodine before every FNAC. Aspiration was done using 21 G, 88 mm long spinal needle attached to a 10 mL disposable syringe through percutaneous and transthoracic approaches. The lesion was identified in exact section by CT scan after the measurement of the site of entry of needle, route of needle, and distance between skin and the lesion on the CT scan monitor. Aspirates were smeared on clean glass slides, wet-fixed, or air-dried and stained by Papanicolaou (Pap), May-Grunwald Giemsa (MGG), and haematoxylin and eosin stains. All patients were watched carefully for the signs of pneumothorax and excessive bleeding. A followup X-Ray was performed four hours after the TTNAC to look for any such signs.

The slides were screened for adequacy of the aspirate and those with inadequate material were not included in the study for calculating results. The cytological diagnoses rendered by full consensus by three experienced cytopathologists were taken as final. The radiological opinion of each individual lesion was also recorded. Both cytological and radiological opinions were tabulated and compared statistically.

## 3. Results

The diagnostic accuracy was 95.94% considering cytological criteria as the standard. Most common age group affected was 4th to 7th decade (83.7%) with a range of 32 years to 77 years. A male preponderance was noted in this study with a M : F ratio of 2.08 : 1.

The predominant lesion found in the present study was malignancy in 63 cases (85.1%), followed by suspicions of malignancy in four cases (5.40%). Two cases each (2.70%) were diagnosed as granulomatous and inflammatory lesions, respectively. The material was inadequate for interpretation in three cases (4%). These cases were omitted from study for further calculations (Table 1).

Out of fifty four FNAC-proven cases of primary neoplasms of lung, forty three were males (79.6%) and eleven were females (20.4%). Hence, there was a significant male preponderance in primary neoplasms of lung with a M : F ratio of 3.9 : 1 (Table 2).

**Figure 1:**
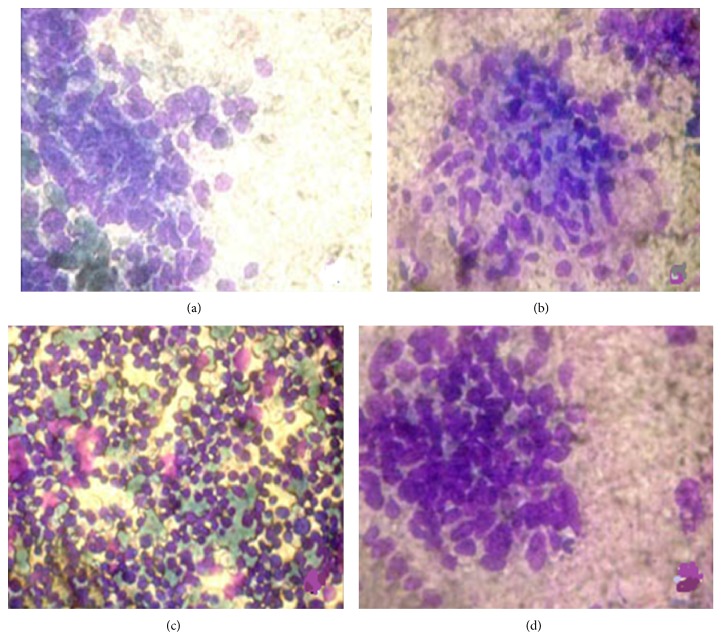
Cytomorphological appearances of various primary lung lesions. (a) Atypical cells of small cell carcinoma exhibiting nuclear moulding and smudging [MGG ×400]. (b) Epithelioid cell collections forming granuloma [MGG ×200]. (c) Monomorphic population of lymphoid cells in lymphoproliferative disorder [MGG ×200]. (d) Atypical cells forming gland pattern in adenocarcinoma [MGG ×400].

On cytological typing of primary lesions, it was observed that the most common malignant lesion seen was adenocarcinoma in twenty four cases (44.5%). Two of these were diagnosed as bronchoalveolar variant of adenocarcinoma. Squamous cell carcinoma was diagnosed as second most common lesion (twenty cases; 37.0%) followed by small cell carcinoma (4 cases; 7.4%) and undifferentiated carcinoma (2 cases; 3.7%). Four cases (7.4%) were reported as suspicious of malignancy owing to paucicellularity (Figure 1). These cases were kept under primary malignant lesions on cytology due to strong clinical and radiological opinion. These lesions could not be subtyped because of aforementioned paucicellularity (Table 2).

A difference in prevalence of primary lung lesions was noted between males versus female subpopulation. While adenocarcinoma was the most common primary lung lesion in females (81%), squamous cell carcinoma was slightly more common than adenocarcinoma in males (18 cases of SCC; 15 cases of adenocarcinoma). Overall, adenocarcinoma, however, was the most common malignancy with a M : F ratio of 1.2 : 1 (Tables 1 and 2).

While tabulating the association of neoplasms with tobacco usage, it was noted that thirty out of fifty patients presenting with primary lung lesions were smokers with all of them being males. Out of these thirty patients, squamous cell carcinoma (16 cases; 53.33%) was the most common malignant subtype followed by adenocarcinoma (9 cases; 30%), small cell carcinoma (4 cases; 13.33%), and undifferentiated carcinoma (1 case; 3.33%). There was, therefore, a strong predisposition to develop lung carcinoma among smokers and squamous cell carcinoma was the most common malignancy among smokers (Table 2).

**Figure 2:**
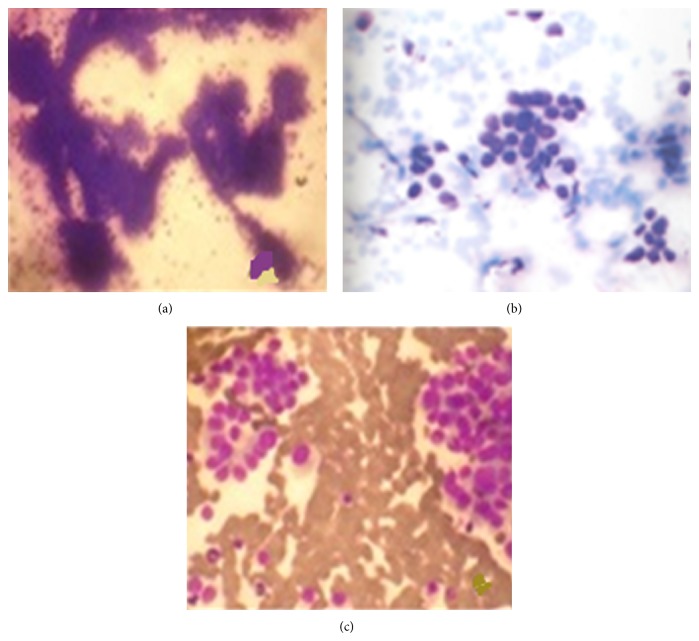
Cytomorphological findings in various metastatic lung lesions. (a) Metastasis of papillary fragments of adenocarcinoma arising from GIT [MGG ×200]. (b) Metastasis of squamous cell carcinoma arising from oral cavity [MGG ×400]. (c) Metastasis of follicular neoplasm thyroid [MGG ×200].

Metastatic deposits (as secondary lung lesions) were seen in six females and four males (M : F ratio—1 : 1.5). In half the cases (50%), in male subjects, the primary lesion was located in oral cavity followed by one case each in vocal cord and gastrointestinal tract (GIT). On the other hand, frequency of primary was equal in female subjects, with 2 cases each metastasizing from breast, GIT, and cervix (Figure 2).

Extrapulmonary lesions from mediastinum were majorly of haematolymphoid origin and all were diagnosed as non-Hodgkin's lymphoma (NHL). One case of mesothelioma in a 50-year-old male was also diagnosed.

No major complications were encountered in this study; however, one patient did complain of dyspnoea following the procedure.

Cytoradiological correlation was also attempted in the present study and it was found to be 89.2%. In 8 cases out of 74, discrepancy was noted owing to 3 cases of inadequate material, 2 cases misdiagnosed as malignant on radiology, and 3 cases wrongly subtyped on radiology.

## 4. Discussion

CT assisted transthoracic FNA procedure not only is minimally invasive yielding quick and accurate diagnosis but also is economical and associated with low morbidity complaints. Other points in favour of FNA over open biopsy and core needle biopsy have high degree of sensitivity and specificity of this procedure in cases of malignancies. A review of thoracic FNA by a study conducted by C. J. R. Stewart and I. S. Stewart [7] revealed a specificity of 100% in cases of malignancies. FNA has also been shown to have a high degree of positive predictive value (99%) in a large study [8]. A negative result, generally, is less reliable and most reports document a false negative rate of 10–20% for lung aspiration cytology. The reported sensitivity and diagnostic accuracy for benign and nonneoplastic lesions are not so high (40–70%) [8–12] prompting many to recommend core needle biopsy to obtain a definitive diagnosis in benign conditions [11–14]. In the present study the male preponderance noted is consistent with the studies done by other researchers [15, 16].

In the study conducted, 84% of cases were diagnosed as non-small cell carcinomas, whereas small cell carcinomas were 12% of the primary lung lesions. This corroborates with the work done by other researchers who have reported an incidence of 70% and 20% in non-small cell and small cell carcinomas, respectively [17, 18].

Adenocarcinoma was the most common malignant lesion followed by squamous cell carcinoma on subtyping the non-small cell carcinomas. This is in concordance with recent work done by other researchers where adenocarcinoma has overtaken squamous cell carcinoma as the leading primary malignant neoplasm [17–19].

In metastatic lesions, adenocarcinoma and squamous cell carcinoma were seen in equal number of cases. The metastatic lesions in cases of males were predominantly located in the oral cavity and larynx which reflects the increased tobacco and alcohol habit in males. Primary tumors in metastatic lesions in females were expectedly located mainly in breast and cervix.

Unlike the work done by other researchers, inadequacy was not a big problem in our study (accounting for 3 cases; 4%) as the study employed the technique of immediate cytological assessment by means of rapid staining procedures and joint presence of both radiologists and pathologists which is recommended by various researchers. The reported rates of inadequacy in other studies range from 16 to 27% [10, 20–23].

Complications of TTFNA reported by various researchers were comprised of pain at the puncture site, pneumothorax, pulmonary haemorrhage, hemoptysis, implantation of the malignant cells in the needle tract, empyema, and bleeding into the chest wall. Pneumothorax is the most common complication with the occurrence rates varying from 3 to 57% with 2–17% requiring a chest tube [24, 25]. In the present study, in only 2 cases, features of mild dyspnoea were noted which resolved within half an hour after procedure. No other significant postprocedural complication was reported.

An attempt was made in the present study to evaluate the degree of agreement between cytology and radiology which was found to be 89.2%. Because cytology was considered as the gold standard, the accuracy of FNA diagnosis is going to be somewhat elevated as compared to studies wherein histology ± immunostaining is used as the gold standard.

Although 100% cytoradiological concordance was noted in cases presenting with metastatic and mediastinal lesions, discordance was noted in five of the primary lung lesions. Three cases of primary malignant lesions of lung which were diagnosed as adenocarcinomas on radiology turned out to be squamous cell carcinomas on cytology. One case of a 60-year-old female, diagnosed as multricentric bronchogenic carcinoma on radiology, turned out to be granulomatous inflammatory kochs pathology on cytology. In the last instance of cytoradiological discrepancy, a reported malignant lesion in the case of a 68-year-old male on radiology turned out to be that of pyogenic abscess when aspirated. Culture growth yielded pseudomonas colonies. The lesion resolved completely with conservative measures. Four cases of lung lesions, which were diagnosed as malignant on radiology, were also reported as highly suspicious of malignancy on cytology. Taking into consideration both radiological and cytological findings, these patients were taken up by treating physicians as cases of primary carcinoma lung.

## 5. Conclusions

The present study thus concludes that CT-guided FNA in thoracic lesions is a simple, safe, and economically prudent technique associated with low morbidity, leading to quick and early diagnosis. Present study also concludes that it leads to an accurate diagnosis in conjunction with CT scan enabling direct visualization leading to greater degree of predicting true positive malignant cases. It should be used as a first line investigation in cases of malignant thoracic lesions. Hence TTFNA of small pulmonary lesions helps in early diagnosis, improved staging, increased chance of effective intervention, and formulating immediate effective management of thoracic mass lesions.

## Figures and Tables

**Table 1:** The demographic description of the study.

Subject	Subheadings	Total number	Percentage
Age	Below 40 years	07	9.46
	40–49 years	15	20.27
	50–59 years	34	45.95
	60–69 years	13	17.56
	70 years and above	05	6.75

Sex	Male	50	67.50
	Female	24	32.50

Type of lesions	Lung mass	71	95.90
	Mediastinal mass	02	2.70
	Pleural mass	01	1.40

History of smoking	Smoker	32	43.20
	Nonsmoker	42	56.80

Radiological diagnosis	Malignant	69	93.20
	Benign	05	06.80

Cytological findings	Malignant	63	85.10
	Suspicious of malignancy	04	05.40
	Benign	04	05.40
	Inadequate	03	04.10

Sampling	Adequate	71	96
	Inadequate	03	04

**Table 2:** Spectrum of lesions in the present study^†^.

Thoracic mass lesions	Cytological diagnosis	Number
Malignant		
1° Lung tumors	Squamous cell carcinoma	20
	Adenocarcinoma	24
	Small cell carcinoma	04
	Large cell carcinoma/undifferentiated	02
	Suspicious of malignancy	04
2° Lung tumors	Metastasis from breast	02
	Metastasis from cervix	02
	Metastasis from GIT	02
	Metastasis from oral cavity	02
	Metastasis from vocal cord	01
	Metastasis from thyroid	01
Mediastinal tumors	Non-Hodgkin's lymphoma	02
Pleural tumors	Mesothelioma	01
Benign	Acute suppurative	02
	Granulomatous	02

^†^Calculations have been done after excluding 03 cases which had inadequate material.
